# Sociodemographic and occupational factors influencing pregnant workers’ awareness and utilization of the New York City Pregnant Workers Fairness Act

**DOI:** 10.1186/s12889-025-23404-w

**Published:** 2025-08-01

**Authors:** Justine Marcinek, Venu Gopal Bangla, Michael Belingheri, Lynn C. Onyebeke, Demetrios M. Papazaharias, Hsiao-Hsien Leon Hsu, Marti D. Soffer, Roberto G. Lucchini, Omara Afzal, Yueh-Hsiu Mathilda Chiu

**Affiliations:** 1 Graduate School of Biomedical Sciences, Department of Public Health, Icahn School of Medicine at Mount Sinai, New York, NY USA; 2 Department of Environmental Medicine, Icahn School of Medicine at Mount Sinai, New York, NY USA; 3 School of Medicine and Surgery, University of Milano-Bicocca, Milan, Italy; 4 Technology Transfer Center, National Cancer Institute, Rockville, MD USA; 5 Department of Obstetrics, Gynecology, and Reproductive Science, Icahn School of Medicine at Mount Sinai, New York, NY USA; 6 Department of Obstetrics, Gynecology and Reproductive Biology, Massachusetts General Hospital, Boston, MA USA; 7 Department of Environmental Health Science, Robert Stempel College of Public Health and Social Work, Florida International University, Miami, FL USA; 8 Department of Obstetrics and Gynecology, Capital Health Medical Center, Trenton, NJ USA; 9 Institute for Exposomic Research, Icahn School of Medicine at Mount Sinai, New York, NY USA

**Keywords:** Pregnant workers, Occupational health, Maternal health, Public health policy, Public health law, Pregnancy

## Abstract

**Background:**

The New York City (NYC) Pregnant Workers Fairness Act (PWFA) went into effect in January 2014 to provide greater flexibility for pregnant workers’ accommodations, yet no studies to date have evaluated its effectiveness and utilization. We examined factors associated with pregnant workers’ PWFA awareness, understanding of PWFA, and receipt of accommodations in a lower socioeconomic status population in NYC.

**Methods:**

Participants included 481 pregnant workers who attended prenatal visits at Mount Sinai Hospital Obstetrics and Gynecology Clinic in NYC in 2017. Detailed demographic and occupational data were collected via in-person interviews using a pregnancy and work survey. Information on participants’ PWFA awareness, knowledge of PWFA-eligible accommodations, and accommodations received was also obtained. Multivariable-adjusted logistic regressions were used to identify the factors influencing PWFA awareness, knowledge of PWFA-eligible accommodations, and accommodations received among six common examples.

**Results:**

Only 14% of participants had ever heard of NYC PWFA legislation. Lower educational level (≤ 12th grade) (Adjusted Odds Ratio [aOR] = 0.54, 95% Confidence Interval [CI] = 0.30–0.98) and being unsure of workplace maternity leave policy (aOR=0.39, 95% CI=0.18-0.87) were associated with lack of PWFA awareness. Regardless of PWFA awareness, no maternity leave policy (aOR = 0.15, 95% CI = 0.04–0.48 vs. paid policy) and being unsure of maternity leave policy (aOR = 0.19, 95% CI = 0.06–0.59 vs. paid policy) were associated with no knowledge of any PWFA-eligible accommodations. Regardless of their PWFA awareness, women working for ≤ 5 years (aOR=0.42, 95% CI=0.22-0.83), non-U.S. born (aOR=0.57, 95% CI=0.36-0.90), and high-risk pregnancy clinic patients (aOR=0.59, 95% CI=0.38-0.93) all had lower odds of receiving PWFA-eligible accommodations, such as adjustment to lighter duty, compared to their counterparts.

**Conclusions:**

Lower educational level, lack of paid workplace maternity leave policy, shorter job tenure, and non-U.S. born were associated with decreased PWFA awareness and/or accommodations received. As more women continue to work during pregnancy, interventions promoting PWFA awareness and utilization are paramount for protecting the maternal and child health of these identified vulnerable groups, especially given that final regulations for a national PWFA recently went into effect across the United States in June 2024.

**Supplementary Information:**

The online version contains supplementary material available at 10.1186/s12889-025-23404-w.

## Introduction

Pregnant workers have become increasingly prevalent in the United States (U.S.) labor force since the 1960s [1]. According to the U.S. Census Bureau, 88% of first-time mothers employed during pregnancy worked into their third trimester, and 65% continued working until less than one month before childbirth [1]. In 2019, approximately two-thirds of women between ages 16–50 who gave birth in a one-year period were in the U.S. labor force [2].

Evidence suggests that prenatal exposures can affect pregnancy-related medical conditions, fetal development, birth outcomes, and the child’s health across the lifespan [3–13]. The prenatal period contains critical windows where both the pregnant woman and the fetus may be more vulnerable to environmental exposures. Physical, chemical, and psychosocial hazards encompass some of the occupational exposures that may impact the health of a pregnant person and their offspring [8]. However, the implementation of protective workplace policies for pregnant workers in the U.S. and pregnant workers’ awareness of these policies remain understudied.

Two landmark amendments to U.S. Public Laws, the Pregnancy Discrimination Act of 1978 (PDA) and the Americans with Disabilities Act Amendments Act of 2008 (ADAAA), are intended to prevent pregnancy-related discrimination and harassment that may occur within various aspects of employment, including hiring, termination, job assignments, employee benefits, employee leave of absence, promotion, and payment [1, 8, 9, 14–19]. Despite these existing legal protections, pregnant workers in the U.S. still face discrimination, including being forced out of their jobs and being denied reasonable accommodations that would enable them to continue working to support their families [9, 15, 16, 20–23]. In 2022, 29.8% of women were the primary income earner for their families, making their ability to work and maintain job benefits throughout pregnancy and parenthood vital for their family’s health and economic stability [15, 17, 24–26]. Pregnant workers may temporarily need reasonable accommodations to work safely [15, 18]. A reasonable accommodation enables a worker to do essential job tasks without placing excessive financial or administrative burden(s) on their employer [15, 19, 26]. Examples include additional bathroom breaks, a revised work schedule, provision of aiding tools or equipment, or temporary adjustment of work assignments to reduce potentially hazardous exposures [19]. However, pregnancy remains a substantial obstacle to women’s ability to secure and maintain employment, particularly in low-wage jobs [15, 16, 20, 24, 27].

Employees who are pregnant, lactating, and/or recovering from a recent birth may have additional rights afforded by other federal, state, or local laws and regulations [14]. In the decades preceding the federal Pregnant Workers Fairness Act (PWFA), thirty states and Washington, D.C. passed laws outlining protections for pregnant workers [8, 10, 28]. The federal PWFA recently went into effect in June 2023, and the Equal Employment Opportunity Commission (EEOC) issued final regulations that became effective in June 2024 [28]. The EEOC is the administrative regulating body responsible for enforcing anti-discrimination law and investigating reports of potential violations [28]. Employers with 15 or more employees fall under the national PWFA and therefore must provide reasonable accommodations in a timely manner upon request [8, 28, 29]. Employees must clearly communicate their limitations to their employers in order to negotiate a reasonable accommodation that fulfills their specific needs. Employees can request different accommodations as their needs change during and after pregnancy. Supporting documentation from health care providers is generally not required to facilitate the negotiation process, though it is permitted in certain circumstances, such as to describe how long a specific accommodation is needed [28].

New York City passed its own PWFA (NYC PWFA) ten years before the federal legislation, which provides a unique opportunity to investigate the effectiveness of the city law before protections were implemented nationwide. The NYC PWFA was passed in October 2013 and has been in effect since January 2014. The New York City Commission on Human Rights enforces New York City Human Rights Law and responds to complaints regarding discrimination in the city. Notably, the NYC PWFA applies to New York City employers with four or more employees, which covers more employers than the federal PWFA [30]. Employers are required to clearly post written notice of PWFA in the workplace and notify new employees about their rights upon hiring [26]. PWFA was enacted to provide better flexibility for workers with pregnancy-related medical conditions so they can work safely. Under NYC PWFA, employees are generally not required to provide medical proof of pregnancy or their pregnancy-related medical condition, and they do not need to prove that their pregnancy-related medical condition qualifies as a disability to request a reasonable accommodation [26]. Employers must provide a clear outline for how to request a reasonable accommodation at work. Workers are strongly encouraged to negotiate with their employer to implement reasonable accommodations that meet their specific needs throughout pregnancy and after childbirth. To our knowledge, although the law has been in effect in New York City for a decade, no studies to date have evaluated PWFA awareness and its effectiveness.

To start addressing these gaps, we conducted a study to assess PWFA recognition and utilization among pregnant working women in the New York City metropolitan area. The objectives of this study were to identify the demographic and occupational factors influencing pregnant workers’ awareness and understanding of the PWFA law. We also examined the factors associated with receiving PWFA-eligible accommodations, regardless of the workers’ PWFA awareness. We aim to provide evidence that can help improve organizational cultures nationwide and better inform intervention strategies for the federal PWFA regulations [28].

## Study Design & Methods

### Study Participants

Participants were from the Effectiveness Study of Pregnant Workers Fairness Act (ESPWFA), a pilot project designed to examine the factors influencing pregnant workers’ recognition and understanding of PWFA in the New York City metropolitan area. We recruited pregnant women who were in the waiting room for their prenatal visits at the Obstetrics and Gynecology (OB/GYN) clinic at Mount Sinai Hospital in Manhattan, New York between January and September 2017, three years after PWFA went into effect in New York City. Eligibility criteria included women 18 years or older, pregnant or within 2 months after childbirth, fluency in English, Spanish, or Mandarin Chinese (the three most common spoken languages in New York City) and having worked in New York City during their current or most recent pregnancy in a workplace with four or more employees since January 2014. Trained research staff administered an anonymous survey on pregnancy and work in the participant’s primary language. These members of the research team were also fluent speakers in their designated survey language. The research staff approached potential participants in the waiting room without knowing or recording their names on the survey. If they were deemed eligible for the study, an ID number was assigned to their survey responses. This ID number was not attached to any identifying information. The survey was set up in the REDCap software system and operated on an iPad, and the participants’ responses were automatically recorded into a secured REDCap (Research Electronic Data Capture) database maintained by the Icahn School of Medicine at Mount Sinai [31, 32]. The participants were not informed about any incentive for participation until they completed the survey (i.e., while they were filling out the survey, they did not know they would receive a $5 gift card for their participation). The study participants were 481 pregnant women who completed all three sections of the pregnancy and work survey, which is described in detail as follows. This survey was developed for the ESPWFA pilot project, and an English language version is provided in Additional File 1, Appendix A. All study procedures were approved by the Institutional Review Board of the Icahn School of Medicine at Mount Sinai.

### Pregnancy and Work Survey

#### Sociodemographic and basic information

Participants reported their basic demographics, including age (in years), race/ethnicity, number of births (parity, categorized into no births until current or most recent pregnancy or *≥* 1 births until current or most recent pregnancy), educational attainment level (categorized into ≤ 12th grade or > 12th grade), marital status (categorized into married/cohabitating or single/divorced/separated/widowed), main language spoken at home (English, Spanish, Mandarin, or Other as specified by the participant), country born (U.S. or other country), whether they were registered as a patient at the high-risk pregnancy clinic (yes or no), and health insurance status. For the analyses, race/ethnicity responses were grouped into Hispanic or Latina (non-Black Hispanic), Black (Black or Hispanic-Black), White (non-Hispanic White), and Other (including Native American, Asian/Pacific Islander, or another race/ethnicity specified by the participant). Health insurance status included the following options as responses: No insurance, Medicaid only, Medicare only, Medicare & Private insurance, Private insurance only, and Other. Of note, some pregnant women qualify for Medicare if they have certain disabilities or significant comorbidities, albeit rarely. To account for low sample sizes in some cells and to distinguish between participants with private insurance from those only covered by public insurance, responses were grouped into the following three categories for analysis: Medicaid only or Medicare only, With private insurance (private insurance only or having both private insurance and Medicare), and No insurance or other/unknown.

#### Occupational/job related information

Participants were asked about the number of jobs they held and part-time/full-time status. Based on their primary job in New York City, they reported job industry/field, whether they were a supervisor/manager, job tenure (grouped into ≤ 5 years or > 5 years), job-related physical activity, occupational exposure to chemicals, job satisfaction, satisfaction with their working relationship with their supervisor/manager, and knowledge of workplace maternity leave policy. Options for job satisfaction and satisfaction with supervisor/manager were both dichotomized into the following categories: ‘Not satisfied at all/Not too satisfied’ and ‘Somewhat satisfied/Very satisfied.’ Knowledge of workplace maternity leave policy was categorized into ‘Yes, paid,’ ‘Yes, unpaid,’ ‘No policy,’ and ‘Unsure of policy.’ Job-related physical activity was classified as high intensity (pushing, pulling, and lifting), moderate intensity (walking and standing), and low intensity (sitting). Participants who answered that they performed more than one type of physical work at their job were grouped into the highest intensity level among the activities reported. Job industry/field was categorized using the National Career Clusters Framework, which is managed by the National Association of State Directors of Career Technical Education Consortium [33]. Career clusters were then collapsed into six categories for data analysis using the Career Fields, Clusters, and Pathways framework, as previously developed [34]. These fields include Agriculture, Food, & Natural Resources; Arts, Communications, & Information Systems; Business, Management, & Administration; Engineering, Manufacturing, & Technology; Health Science Technology; and Human Services [34]. Women whose job was classified as ‘Restaurants and Food/Beverage Services’ were reclassified into the Human Services job sector if they were not a supervisor/manager at their worksite. To measure occupational exposure to chemicals, participants were first asked whether they were exposed to any chemicals in the workplace before their current or most recent pregnancy and during their current or most recent pregnancy. If they answered ‘yes’ to either of these questions, they were asked to check all that apply among the following exposures: cleaning chemicals, pesticides, paints/sprays, metals, and other chemical(s), of which they were asked to specify. They were then asked how many hours per day they were exposed to these chemicals.

#### Awareness and understanding of PWFA law and accommodations

The last section of the survey assessed participants’ familiarity with, and understanding of, accommodations covered by PWFA and requirements for eligible employers. Detailed structure of the questions is shown in Additional File 1. Participants were first asked whether they had heard of PWFA (yes or no). If they answered ‘yes,’ they were asked to indicate how they heard about PWFA. Participants were then asked if they knew what benefits were covered by PWFA (yes or no). If they answered ‘yes,’ they were subsequently asked to select all of the commonly requested PWFA-eligible accommodations they were aware of among the following six examples: adjustment to lighter duty (e.g., help with lifting, temporary transfer to a less physically demanding job), breaks to drink water, occasional breaks to rest, time off for recovery from childbirth, changes to the work environment (e.g., removal of toxic chemicals), and a modified work schedule (e.g., temporarily changing work shifts). Of note, we selected these six examples a priori in our survey as they are among the most common reasonable accommodations requested by pregnant workers, though individuals can negotiate other accommodations with their employer to fit their specific needs at work. Participants were then asked to select all of the commonly requested PWFA-eligible accommodations they had ever received at work during their pregnancy, regardless of their awareness of the PWFA law. Participants also reported whether they were aware that their employer is required by law to provide reasonable accommodations during and/or after pregnancy.

### Statistical Analyses

Distributions of participants’ sociodemographic and occupational factors were calculated. We also calculated frequency of PWFA awareness and the number of PWFA-eligible accommodations participants recognized among the six examples. We then conducted univariate and multivariable-adjusted models to examine the factors associated with pregnant workers’ (1) PWFA awareness, (2) knowledge of PWFA-eligible accommodations, and (3) receipt of PWFA-eligible accommodations regardless of PWFA awareness. All three model types utilized logistic regression for binary outcome variables (i.e., yes vs. no). First, we used logistic regression to identify the factors associated with PWFA awareness (i.e., ever vs. never heard of PWFA, Model Type (1)). Next, we used logistic regression to examine the factors influencing whether pregnant workers knew any accommodations covered by the PWFA law (Model Type (2)). Additionally, to examine whether any factors were associated with the number of accommodations recognized by participants among the six provided, we also conducted zero-inflated Poisson regression for discrete count data ranging from 0 to 6, given that 92.5% of participants did not recognize any accommodations covered by the PWFA law. Furthermore, pregnant workers may have already received eligible accommodations even though they never heard of the PWFA law. Therefore, we also considered whether participants had received any of the aforementioned PWFA-eligible accommodations, regardless of their PWFA awareness, as additional outcomes of interest (Model Type (3)). We conducted separate logistic regression models to identify factors associated with receipt of each PWFA-eligible accommodation.

Odds ratios and corresponding 95% confidence intervals (95% CI) are reported as primary measures of association for logistic regressions. To enhance interpretability of results, we also estimated the marginal effects of these logistic regression models using methods from previous studies [35, 36]. In brief, after fitting the logistic regression model, we estimated the marginal effect by shifting the exposure variable between reference and comparison values, while holding other covariates at their observed levels, and calculated the average change in predicted probability. Confidence intervals were generated using bootstrap resampling with 2000 iterations to account for variability in the estimates. For all multivariable-adjusted models, we considered standard control variables (e.g., age, race/ethnicity, educational attainment level) as covariates. In addition, we considered a list of variables (e.g., marital status, supervisor/manager status, parity, job satisfaction, satisfaction with supervisor/manager, country born, job tenure, knowledge of maternity leave policy, health insurance, high-risk pregnancy clinic patient status) as potential covariates. Variables that were significant or approaching significance with *p*≤0.10 in their respective univariate models were considered as covariates in the multivariable models. All statistical analyses were conducted using SAS OnDemand for Academics software [37].

## Results

The sociodemographic characteristics of participants are summarized in Table 1. Participants were primarily from racial/ethnic minority groups, with approximately 34.5% identifying as Black and 48.7% identifying as Hispanic. Approximately half (49.3%) had completed a high school education at most; 33.9% were born outside of the U.S., and 75.1% had Medicaid.

**Table 1:** Sociodemographic characteristics of study participants (*n* = 481)

Variable	Distribution
**Age (years) [mean (SD)]**	28.8 (5.96)
**Race and Ethnicity [n (%)]**	
Non-Hispanic White	31 (6.4)
Black/Hispanic-Black	166 (34.5)
Non-Black Hispanic	234 (48.7)
Other^**a**^	50 (10.4)
**Educational Level [n (%)]** ^**b**^	
≤ 12th grade	236 (49.3)
> 12th grade	243 (50.7)
**Maternity leave policy [n (%)]**	
Paid	128 (26.6)
Unpaid	98 (20.4)
No policy	115 (23.9)
Not sure	140 (29.1)
**Job tenure [n (%)]**	
5 years or less	432 (89.8)
More than 5 years	49 (10.2)
**Main language spoken at home [n (%)]**	
English	350 (72.8)
Spanish	97 (20.2)
Mandarin or Other	34 (7.1)
**Country Born [n (%)]**	
United States (U.S.)	318 (66.1)
Other	163 (33.9)
**Health insurance [n (%)]**	
No insurance	8 (1.7)
Medicaid only	361 (75.1)
Medicare only	26 (5.4)
Medicare & Private	14 (2.9)
Private	44 (9.2)
Other/Unknown	28 (5.8)
**Parity [n (%)]**	
0 births besides current/recent pregnancy	192 (39.9)
1 + births besides current/recent pregnancy	289 (60.1)
**Supervisor/manager status [n (%)]**	
No	395 (82.1)
Yes	86 (17.9)
**High-risk pregnancy clinic patient [n (%)]** ^**c**^	
No	353 (73.9)
Yes	125 (26.2)
**Job industry [n (%)]**	
Business, Management, & Administration	98 (20.4)
Agriculture, Food, & Natural Resources	52 (10.8)
Arts, Communications, Information Systems or Human Services	166 (34.5)
Health Science Technology	102 (21.2)
Engineering, Manufacturing, & Technology	21 (4.4)
Other	42 (8.7)

^a^ Native American, Asian/Pacific Islander, or another race/ethnicity specified by the participant; ^b^ Missing 2 observations, *n =* 479; ^c^ Missing 3 observations, *n =* 478

### PWFA awareness

Among the total sample of 481 participants, only 14% (*n* = 67) reported that they had ever heard of PWFA; *n* = 28 (6% of total sample) were informed by a current or past employer, and *n* = 39 (8% of total sample) heard about it from another source (e.g., the internet, family/friends, or a PWFA brochure).

Table 2 presents the results from logistic regression models examining the factors that influenced pregnant workers’ awareness of the PWFA law. The results from univariate models and multivariable-adjusted models were generally consistent. After adjusting for covariates in the multivariable analysis, those with a lower educational level (≤12th grade) had 46% lower odds of recognizing PWFA compared to those who attained higher education (adjusted OR [aOR] = 0.54, 95% CI: 0.30, 0.98, *p* = 0.04). Compared to women who reported having a paid maternity leave policy, women who were unsure of their workplace’s maternity leave policy had 61% lower odds of recognizing PWFA (aOR = 0.39, 95% CI: 0.18, 0.87, *p* = 0.02). Other variables were not significantly associated with PWFA awareness in both univariate and multivariable models.

**Table 2:** Univariate and multivariable-adjusted logistic regression models examining the sociodemographic and occupational factors associated with PWFA awareness

Characteristic	Univariate Model^a^		Multivariable-adjusted Model^b^		
	Odds Ratio (95% CI)	*p*-value	Adjusted Odds Ratio (95% CI)	*p*-value	Estimated Marginal Effect^c^
**Age (years)**	0.98 (0.94, 1.02)	0.35	0.98 (0.93, 1.03)	0.34	-0.3% (-0.9%, 0.3%)
**Race/Ethnicity**					
White	Ref.	--	Ref.	--	Ref.
Black	2.45 (0.55, 10.95)	0.24	2.31 (0.49, 10.92)	0.29	7.2% (-5.1%, 16.7%)
Hispanic	2.13 (0.48, 9.40)	0.32	2.14 (0.46, 9.96)	0.33	6.3% (-5.5%, 14.9%)
Other	4.09 (0.84, 19.88)	0.08	3.92 (0.76, 20.27)	0.1	14.2% (-0.2%, 28.7%)
**Education**					
>12th grade	Ref.	--	Ref.	--	Ref.
≤12th grade	0.53 (0.31, 0.90)	0.02	0.54 (0.30, 0.98)	0.04	-6.7% (-12.8%, -0.2%)
**Maternity leave policy**					
Paid	Ref.	--	Ref.	--	Ref.
Unpaid	1.17 (0.60, 2.28)	0.64	1.21 (0.60, 2.45)	0.59	2.8% (-8.7%, 13.9%)
No policy	0.53 (0.25, 1.13)	0.1	0.53 (0.24, 1.16)	0.11	-7.3% (-16.4%, 1.0%)
Unsure	0.43 (0.20, 0.90)	0.03	0.39 (0.18, 0.87)	0.02	-9.8% (-18.4%, -2.0%)

^a^ For age, race/ethnicity, education, and maternity leave policy univariate models, *n* = 480; for education univariate model, *n* = 479

^b^ Model adjusted for all characteristics shown in table, as well as marital status, job satisfaction, satisfaction with supervisor/manager, country born, job tenure, health insurance, and high-risk pregnancy clinic patient status; *n* = 476

^c^ The marginal effect represents the average change in predicted probability of the outcome under a counterfactual shift of the predictor from its reference value, holding all other covariates at their observed values. Confidence intervals were calculated via bootstrapping with 2000 iterations, based on the empirical distribution of marginal effects across repeated model estimations [35, 36]

### Knowledge of PWFA accommodations

Participants who reported having heard of PWFA (*n* = 67) were additionally asked to select all of the PWFA-eligible accommodations they recognized out of the six examples listed. Over half of respondents (57%) indicated that they did not know any of these accommodations were covered by PWFA, while 16% recognized one to four accommodations, and 27% recognized five or more accommodations among the list.

Table 3 presents the results from univariate and multivariable-adjusted logistic regression models examining the factors associated with whether participants knew any PWFA-eligible accommodations (yes vs. no). Results from both univariate and multivariable models suggested that knowledge of workplace maternity leave policy is most associated with recognizing PWFA accommodations. Compared to women whose workplace had a paid maternity leave policy, women whose workplace did not have a maternity leave policy had lower odds of knowing any accommodations after adjusting for covariates (aOR = 0.15, 95% CI: 0.04, 0.48, *p* = 0.002), with similarly low odds among women who were unsure of their workplace maternity leave policy (aOR = 0.19, 95% CI: 0.06, 0.59, *p* = 0.004). For women whose job had an unpaid maternity leave policy, the odds of not knowing PWFA-eligible accommodations were also lower compared to women whose job had a paid maternity leave policy, though not statistically significant. High-risk pregnancy clinic patient status was also a suggestive predictor, though not statistically significant. In comparison to managers, non-managers had lower odds of knowing any PWFA accommodations for both univariate and multivariable models (aOR = 0.34, 95% CI: 0.15, 0.76, *p* = 0.01). Other variables such as job tenure, job satisfaction, satisfaction with supervisor/manager, country born, and marital status were not significantly associated with knowledge of PWFA-eligible accommodations. We also conducted a zero-inflated Poisson model examining the factors associated with the number of PWFA-eligible accommodations recognized by participants (i.e., discrete count of PWFA-eligible accommodations recognized among the six examples, ranging from 0 to 6). Given that 92.5% of participants did not recognize any PWFA-eligible accommodations, the logit component of the zero-inflated Poisson model yielded similar results and conclusions as the multivariable logistic regression model presented in Table 3; the count (Poisson) component of the model suggested that none of the factors were significantly associated with the number of accommodations recognized among non-zero respondents (see Supplemental Table S1 in Additional File 1).

**Table 3:** Univariate and multivariable-adjusted logistic regression models examining the sociodemographic and occupational factors associated with whether participants knew any PWFA accommodations (yes vs. no)

Characteristic	Univariate Model ^a^		Multivariable-adjusted Model ^b^		
	Odds Ratio (95% CI)	*p*-value	Adjusted Odds Ratio (95% CI)	*p*-value	Estimated Marginal Effect ^c^
**Age**	1.03 (0.97, 1.09)	0.33	1.03 (0.97, 1.11)	0.33	0.2% (-0.4%, 0.6%)
**Race/Ethnicity**					
White	Ref.	--	Ref.	--	Ref.
Black & Hispanic	0.69 (0.30, 1.57)	0.38	0.89 (0.34, 2.35)	0.82	-0.7% (-8.7%, 5.5%)
**Education**					
>12th grade	Ref.	--	Ref.	--	Ref.
≤12th grade	0.56 (0.27, 1.12)	0.1	0.63 (0.29, 1.35)	0.23	-2.9% (-7.2%, 2.3%)
**Maternity leave policy**					
Paid	Ref.	--	Ref.	--	Ref.
Unpaid	0.48 (0.20, 1.14)	0.1	0.54 (0.22, 1.34)	0.18	-6.0% (-14.9%, 2.3%)
No policy	0.20 (0.06, 0.59)	0.004	0.15 (0.04, 0.48)	0.002	-12.2% (-19.8%, -6.2%)
Unsure	0.16 (0.05, 0.48)	0.001	0.19 (0.06, 0.59)	0.004	-11.5% (-19.0%, -5.1%)
**High-risk pregnancy clinic**					
No	Ref.	--	Ref.	--	Ref.
Yes	0.56 (0.23, 1.39)	0.21	0.50 (0.19, 1.31)	0.16	-3.9% (-8.4%, 0.8%)
**Supervisor/manager status**					
Yes	Ref.	--	Ref.	--	Ref.
No	0.40 (0.19, 0.84)	0.01	0.34 (0.15, 0.76)	0.01	-8.5% (-16.7%, -0.9%)

^a^ For age, race/ethnicity, maternity leave policy, and supervisor/manager status univariate models, *n* = 480; for education univariate model, *n* = 478; for high-risk pregnancy clinic patient univariate model, *n* = 477

^b^ Multivariable model adjusted for all characteristics shown in table, as well as marital status, job satisfaction, satisfaction with supervisor/manager, country born, and job tenure; *n* = 475

^c^ The marginal effect represents the average change in predicted probability of the outcome under a counterfactual shift of the predictor from its reference value, holding all other covariates at their observed values. Confidence intervals were calculated via bootstrapping with 2000 iterations, based on the empirical distribution of marginal effects across repeated model estimations [35, 36]

We also examined whether women knew that their employer was required by law to provide reasonable accommodations during and/or after pregnancy (yes vs. no). The multivariable logistic regression model was adjusted for age, race/ethnicity, educational attainment level, marital status, job satisfaction, satisfaction with supervisor/manager, country born, insurance status, PWFA awareness, high risk pregnancy clinic patient status, and job tenure. We found that women whose workplace had no maternity leave policy (aOR = 0.46, 95% CI: 0.25, 0.85, *p* = 0.01) or women unsure of their maternity leave policy (aOR = 0.54, 95% CI: 0.31, 0.97, *p* = 0.04) had lower odds of knowing about this requirement than women whose job had a paid maternity leave policy. Compared to women with Medicaid only or Medicare only, women with private health insurance had higher odds of recognizing this requirement (aOR = 1.93, 95% CI: 1.03, 3.62, *p* = 0.04). The odds of high-risk pregnancy clinic patients knowing this requirement were lowered by 53% relative to non-high risk patients (aOR = 0.47, 95% CI: 0.28, 0.80, *p* = 0.005). Moreover, pregnant workers who were ‘not satisfied at all’ or ‘not too satisfied’ with their job had significantly lower odds of recognizing this requirement than those who were ‘somewhat satisfied’ or ‘very satisfied’ with their job (aOR = 0.29, 95% CI: 0.15, 0.58, *p* = 0.0004).

### Receipt of PWFA-eligible accommodations, regardless of PWFA awareness

Regardless of PWFA awareness, respondents were asked whether they received any accommodations from the list of six common PWFA-eligible accommodations. Table 4 summarizes the results from separate multivariable logistic regressions identifying the factors associated with receipt of each type of accommodation, regardless of PWFA awareness.

**Table 4:** Multivariable-adjusted logistic regression models examining the sociodemographic and occupational factors associated with PWFA-eligible accommodations received, regardless of PWFA awareness*

Accommodations Received Predictors	Adjustment to lighter duty		Breaks to drink water		Time off for childbirth recovery		Change to work environment		Modified work schedule		Breaks to rest	
	aOR (95% CI)	*p*-value	aOR (95% CI)	*p*-value	aOR (95% CI)	*p*-value	aOR (95% CI)	*p*-value	aOR (95% CI)	*p*-value	aOR (95% CI)	*p*-value
**Job tenure**												
>5 years	Ref.	--	Ref.	--	Ref.	--	Ref.	--	Ref.	--	Ref.	--
≤5 years	0.42 (0.22, 0.83)	0.01	0.47 (0.24, 0.92)	0.03	0.18 (0.09, 0.38)	< 0.0001	0.23 (0.11, 0.48)	< 0.0001	0.46 (0.23, 0.89)	0.02	0.45 (0.23, 0.86)	0.02
**Maternity leave policy**												
Paid	Ref.	--	Ref.	--	Ref.	--	Ref.	--	Ref.	--	Ref.	--
Unpaid	0.56 (0.32, 1.00)	0.05	0.66 (0.38, 1.16)	0.15	0.45 (0.24, 0.82)	0.01	0.66 (0.31, 1.38)	0.27	0.66 (0.36, 1.20)	0.17	0.67 (0.37, 1.20)	0.18
No policy	0.29 (0.16, 0.52)	< 0.0001	0.47 (0.27, 0.81)	0.006	0.30 (0.16, 0.55)	0.0001	0.36 (0.17, 0.80)	0.01	0.54 (0.30, 0.98)	0.04	0.59 (0.34, 1.05)	0.07
Unsure	0.56 (0.33, 0.96)	0.04	0.44 (0.26, 0.74)	0.002	0.15 (0.08, 0.28)	< 0.0001	0.52 (0.25, 1.05)	0.07	0.38 (0.21, 0.69)	0.001	0.53 (0.31, 0.92)	0.02
**Health insurance**												
Medicaid only or Medicare only	Ref.	--	Ref.	--	Ref.	--	Ref.	--	Ref.	--	Ref.	--
No insurance or unknown	1.29 (0.60, 2.79)	0.51	1.05 (0.50, 2.19)	0.90	0.88 (0.35, 2.16)	0.77	1.00 (0.35, 2.83)	1.00	0.96 (0.41, 2.27)	0.93	1.93 (0.90, 4.13)	0.09
With private insurance ^a^	2.30 (1.24, 4.26)	0.008	1.70 (0.93, 3.11)	0.09	3.24 (1.69, 6.20)	0.0004	1.00 (0.44, 2.28)	0.99	2.52 (1.36, 4.67)	0.003	2.39 (1.29, 4.43)	0.006
**Country born**												
United States (U.S.)	Ref.	--	Ref.	--	Ref.	--	Ref.	--	Ref.	--	Ref.	--
Other country	0.57 (0.36, 0.90)	0.02	0.68 (0.44, 1.05)	0.08	0.60 (0.36, 1.01)	0.05	0.97 (0.53, 1.77)	0.91	0.96 (0.59, 1.55)	0.85	0.46 (0.29, 0.74)	0.001
**Satisfaction with supervisor**												
Very satisfied/ Somewhat satisfied	Ref.	--	Ref.	--	Ref.	--	Ref.	--	Ref.	--	Ref.	--
Not too satisfied/ Not satisfied at all	0.33 (0.16, 0.70)	0.004	0.67 (0.35, 1.29)	0.23	0.59 (0.25, 1.39)	0.23	0.53 (0.18, 1.58)	0.25	0.48 (0.20, 1.12)	0.09	0.40 (0.20, 0.84)	0.01
**Satisfaction with job**												
Very satisfied/ Somewhat satisfied	Ref.	--	Ref.	--	Ref.	--	Ref.	--	Ref.	--	Ref.	--
Not too satisfied/ Not satisfied at all	0.69 (0.39, 1.22)	0.21	0.64 (0.37, 1.09)	0.10	0.38 (0.19, 0.78)	0.01	0.79 (0.35, 1.80)	0.58	0.66 (0.34, 1.27)	0.21	0.88 (0.50, 1.55)	0.65
**Marital status**												
Married/cohabitating	Ref.	--	Ref.	--	Ref.	--	Ref.	--	Ref.	--	Ref.	--
Single/divorced/ separated/widowed	0.89 (0.58, 1.36)	0.59	0.77 (0.51, 1.16)	0.21	0.74 (0.46, 1.20)	0.22	0.78 (0.44, 1.39)	0.40	0.56 (0.35, 0.90)	0.02	0.70 (0.45, 1.07)	0.10
**High-risk pregnancy clinic**												
No	Ref.	--	Ref.	--	Ref.	--	Ref.	--	Ref.	--	Ref.	--
Yes	0.83 (0.52, 1.33)	0.45	0.59 (0.38, 0.93)	0.02	0.60 (0.35, 1.03)	0.06	0.55 (0.28, 1.11)	0.09	0.84 (0.50, 1.41)	0.51	0.50 (0.30, 0.82)	0.006

*All models (*n* = 476) are adjusted for age, race/ethnicity group, educational attainment level, PWFA awareness, and all variables listed in the table

^a^ Including participants who only have private insurance or have both private insurance and Medicare

Job tenure (≤ 5 years vs. >5 years) was significantly associated with all six outcomes (*p* ≤ 0.03), where women working for ≤ 5 years at their current job had significantly lower odds of receiving adjustment to lighter duty (aOR = 0.42, 95% CI: 0.22, 0.83, *p* = 0.01), breaks to drink water (aOR = 0.47, 95% CI: 0.24, 0.92, *p* = 0.03), time off for childbirth recovery (aOR = 0.18, 95% CI: 0.09, 0.38, *p* < 0.0001), changes to work environment (aOR = 0.23, 95% CI: 0.11, 0.48, *p* < 0.0001), modified work schedule (aOR = 0.46, 95% CI: 0.23, 0.89, *p* = 0.02), and breaks to rest (aOR = 0.45, 95% CI: 0.23, 0.86, *p* = 0.02). Maternity leave policy was also significantly or borderline significantly associated with all six outcomes, wherein women unsure of maternity leave policy and women without a maternity leave policy had lower odds of receiving reasonable accommodations (*p* = 0.07 for breaks to rest and change to the work environment, *p* ≤ 0.04 for all other accommodations) compared to those whose workplace had a paid maternity leave policy. Women who were high-risk pregnancy clinic patients had significantly lower odds of receiving breaks to rest (aOR = 0.50, 95% CI: 0.30, 0.82, *p* = 0.006) and breaks to drink water (aOR = 0.59, 95% CI: 0.38, 0.93, *p* = 0.02) than women who were not high-risk patients. Compared to women with Medicaid only or Medicare only, women with private health insurance or both Medicare & private insurance had higher odds of receiving four types of accommodations (*p* < 0.01 for adjustment to lighter duty, time off for childbirth recovery, modified work schedule, and breaks to rest). Pregnant workers born outside of the U.S. had 54% lower odds of receiving breaks to rest than U.S.-born women (aOR = 0.46, 95% CI: 0.29, 0.74, *p* = 0.001). The odds of workers born outside the U.S. receiving lighter duty were also significantly lower than U.S.-born workers (aOR = 0.57, 95% CI: 0.36, 0.90, *p* = 0.02). Women who were ‘not satisfied at all’ or ‘not too satisfied’ with their working relationship with their supervisor/manager had significantly lower odds of receiving lighter duty (aOR = 0.33, 95% CI: 0.16, 0.70, *p* = 0.004) and breaks to rest (aOR = 0.40, 95% CI: 0.20, 0.84, *p* = 0.01) than women who were ‘somewhat satisfied’ or ‘very satisfied’ with this relationship. Women who were ‘not satisfied at all’ or ‘not too satisfied’ with their job had lower odds of receiving time off for childbirth recovery than women who were ‘somewhat satisfied’ or ‘very satisfied’ with their job (aOR = 0.38, 95% CI: 0.19, 0.78, *p* = 0.01). Single, divorced, separated, or widowed women had lower odds of obtaining a modified work schedule than married or cohabitating women (aOR = 0.56, 95% CI: 0.35, 0.90, *p* = 0.02).

## Discussion

To our knowledge, this is the first study to assess the sociodemographic and occupational factors associated with pregnant workers’ awareness and understanding of the New York City Pregnant Workers Fairness Act (NYC PWFA). We found that educational level and knowledge of maternity leave policy were significant predictors of PWFA awareness. Knowledge of maternity leave policy and supervisor/manager status were significant predictors for recognition of PWFA-eligible accommodations. Furthermore, regardless of participants’ awareness of the PWFA law, shorter job tenure, being born outside of the U.S., lack of paid maternity leave policy, lack of private health insurance, and being a patient at the high-risk pregnancy clinic were significantly associated with lower odds of receiving PWFA-eligible accommodations at work.

Educational attainment level was a significant predictor of PWFA awareness. Education is a widely used proxy indicator of socioeconomic status, and pregnant workers who obtained higher education may be in professions and/or social networks where knowledge about PWFA was readily available and shared. Marti-Castaner et al. found that new mothers in NYC obtained low-wage jobs in order to meet their immediate needs for income, housing, and welfare; this came at the cost of forgoing higher education [22]. In a study of maternal protective legislation in the Netherlands, van Beukering et al. found that compared to pregnant workers who completed higher vocational education or university, pregnant workers with lower educational attainment were among the most vulnerable groups at risk for working in unsuitable conditions that did not meet legislative guidelines [38]. Mothers with lower educational attainment are more likely to leave their jobs, which may suggest a lack of familiarity with their rights as employees [1, 39].

It is important to target lower socioeconomic status populations and new hires when planning interventions to promote PWFA awareness. Job tenure was significantly associated with receipt of PWFA-eligible accommodations regardless of respondents’ awareness and knowledge of the law. Workers who have been at their job longer may be more familiar with their supervisors/managers, or may be a supervisor/manager themselves, and thus may be more comfortable advocating for and implementing accommodations. They may have had more time to familiarize themselves with their rights as workers and thus can provide additional support to other employees navigating the cooperative dialogue process with their employer. Modeling the use of workplace accommodations can also promote a more supportive work culture, inspiring others to initiate negotiations for reasonable accommodations should the need arise.

We found that satisfaction with one’s supervisor/manager was a significant predictor of receiving PWFA-eligible accommodations. The presence of supportive supervisors/managers could reduce pregnancy-related discrimination by promoting PWFA awareness and encouraging use of reasonable accommodations. In a recent analysis of pregnancy discrimination charges filed with the U.S. Equal Employment Opportunity Commission, McCann & Tomaskovic-Devey found that firms with more women in management positions were generally less likely to be charged with pregnancy discrimination [16]. Managers who received training on family-supportive policies and practices could reduce turnover intentions and promote job satisfaction among their employees [40]. Moreover, pregnant employees who perceived higher levels of social support may experience fewer symptoms of mental health distress [41].

Knowledge of maternity leave policy consistently had statistically significant associations with various outcomes, including pregnant workers’ awareness of PWFA, knowledge of common PWFA-eligible accommodations, receipt of PWFA-eligible accommodations, and awareness of their employer’s legal obligation to provide accommodations. Relative to those whose workplace did not have a paid maternity leave policy (i.e., no policy or unpaid policy only) or who were unsure of their maternity leave policy, women with a paid maternity leave policy might have done additional research into their rights as a pregnant worker and learned how to exercise them. When PWFA went into effect in 2014, only 13% of civilian workers in the U.S. had paid family leave (defined as leave to care for a newborn child, a newly adopted or fostered child, or a seriously ill relative) [42]. When we collected study data in 2017, that percentage had risen to 15% [42]. To date, the U.S. does not have a federal law for paid family leave [17, 18, 20, 27, 43, 44]. Nationally, access to paid family leave is less common for workers in low-wage or part-time jobs, smaller organizations, and underrepresented racial/ethnic groups [16, 22, 27, 44, 45]. It can be challenging to navigate what options are available for leave, driving employees to seek additional guidance and support [23, 44]. Although up to 12 weeks of unpaid leave is available to qualifying employees with the Family Medical Leave Act of 1993, mothers are often ineligible or cannot afford to use it, especially those who are in racial/ethnic minority groups, low-income, less educated, or unmarried [1, 8, 17, 23, 27, 39, 43–45]. This can result in excessive use of sick leave. In France, Henrotin et al. found that pregnant employees used sick leave (thereby accepting reduced pay) when reasonable accommodations might have been more suitable for their needs [46]. Abderhalden-Zellweger et al. posited that pregnant workers in French-speaking Switzerland may request sick leave from their OBGYNs in order to mitigate potential conflict with their employers [47]. Of note, starting January 2018, New York State policy granted eligible employees the right to take up to 12 weeks of job-protected, paid family leave with continued healthcare coverage under their current employer [48, 49]. Our study was conducted before this paid family leave policy was fully implemented in New York State. This state policy warrants future studies, as it might promote awareness of the PWFA law.

Over half of all women who reported being aware of PWFA were not aware of possible accommodations, though employers are responsible for informing their employees about PWFA and pregnant workers’ rights in accordance with NYC Human Rights Law [26]. Still, research suggests that laws and policies protecting pregnant workers are not always fully implemented in practice [6]. In the Netherlands, van Beukering et al. found that merely 15% of employers properly informed pregnant workers of their right to accommodations in compliance with national legislation [38].

Our analyses also reveal that some participants still received PWFA-eligible accommodations even without awareness or understanding of the PWFA law. The odds of receiving these accommodations were affected by several occupational and demographic factors, one of which being health insurance. Our findings suggest that employees with private health insurance had higher odds of receiving accommodations than those without private insurance, even after controlling for educational attainment. Perhaps these employees secured accommodations to continue working as long as possible, avoid loss of earnings, and maintain employer-sponsored health insurance coverage for themselves and their dependent family members [17]. The significant findings of health insurance status in our analyses may be partially explained by its relationship to job type. Job industry could influence what options are available for health insurance. However, we did not find consistently significant associations between job type/industry and the PWFA-related outcomes in our data, possibly due to limited sample size in some job categories. Future studies with larger sample sizes should comprehensively explore the role of job type/industry in the association between health insurance status and PWFA awareness/effectiveness. Some jobs may have the flexibility to provide a wider range of accommodations; employees can engage in a cooperative dialogue with their employers to find accommodations that suit their individual needs.

Notably, pregnant employees who identified their marital status as ‘single, divorced, separated, or widowed’ had lower odds of receiving the ‘modified work schedule’ outcome than those who were married or cohabitating. This could indicate that a pregnant employee who is single may forgo modifying their work schedule (e.g., switching shifts) because they do not have another earner in the household who can compensate and mitigate the potential impact of wages lost [39].

Workers born outside of the U.S. had significantly lower odds of receiving accommodations compared to native-born workers. The ‘country born’ variable was a significant or borderline significant predictor for receipt of lighter duty, breaks to drink water, time off for childbirth recovery, and breaks to rest. Immigrant workers are overrepresented in low-wage jobs that often require physical labor (e.g., food service, domestic & commercial cleaning) [21, 29]. Given that efficiency and productivity are especially valued in these types of fast-paced jobs, workers and employers may hesitate to negotiate accommodations that modify job responsibilities, attendance, and how time is spent at work [21, 23, 24]. Research concentrated on Hispanic & Latina immigrant workers in the U.S. demonstrates that they are at risk of labor exploitation, but may not seek legal protections because they do not know their rights and/or fear harassment, retaliation, or conflicts with the law [21, 24, 29]. These concerns are exacerbated during pregnancy, especially when the expectant mother is undocumented [24]. Though we did not evaluate citizenship/immigration status in our study, this demographic of workers could benefit from targeted outreach about PWFA.

Status as a high-risk pregnancy clinic patient did not make a significant difference in PWFA awareness. However, the odds of high-risk pregnancy clinic patients receiving accommodations were lower than pregnant workers who were not high-risk patients. This is alarming, as one might expect high-risk patients to be more informed about reasonable accommodations due to their susceptibility to medical complications during pregnancy, and accommodations could reduce their risk of work-related illness or injury [8, 22, 23]. Furthermore, one might expect high-risk patients to be more informed about accommodations from their own research, recommendations from their medical care team, their employers, or word of mouth from family/friends/colleagues [18, 23, 50]. Future interventions could provide health practitioners with more educational resources, trainings, or communication platforms about employees’ rights and PWFA, given that pregnant workers with high-risk pregnancies could particularly benefit from accommodations [6, 15, 50].

This study has several strengths. First, the survey was offered in three of the most commonly spoken languages in NYC to capture a broader sample of pregnant workers in the NYC metropolitan area [51]. Moreover, the survey was administered in person; thus, the research staff could directly clarify any questions a participant might have had and communicate in the language the participant was most comfortable speaking. Furthermore, this study is the first to examine the effectiveness of the New York City PWFA through the lens of employees. Our findings suggest that within the first five years of its enactment, the New York City PWFA was considerably underutilized. PWFA is only effective if the individuals it is intended to benefit are fully informed about their rights and feel comfortable exercising them in the workplace without fear of harassment or retaliation. Our study identified several important sociodemographic and occupational factors that could inform future intervention strategies. Interventions can disseminate information about PWFA in various ways. For example, in Japan, Wada et al. developed the Job Adjustment Mobile App, a smartphone application that recommended action plans for pregnant workers to implement job accommodations based on their specific needs, national legislative guidelines, and other pertinent resources [52]. Clear communication is key to ensuring that PWFA is effective, especially for the vulnerable populations identified in our study.

Additional research attests that workplace accessibility benefits all. According to a survey administered to employers by the Job Accommodation Network, 56% of respondents stated that it cost $0 to incorporate accommodations into the workplace. Provision of reasonable accommodations reduces employee attrition and absence, and is more cost-effective than onboarding a new employee [20, 53]. Investing in employees’ safety and comfort stimulates productivity and increases job satisfaction, all while saving businesses money on workers’ compensation and insurance [15, 20, 53]. These findings collectively suggest that promoting awareness and implementation of the PWFA law may benefit employees and employers alike, as supportive workplaces uplift workers and businesses.

We also acknowledge some limitations of this study. As with all surveys, the survey instrument used in our study is subject to recall bias and recency bias. How far along a participant was at the time of the survey could have affected their perception of accommodations (i.e., whether certain accommodations were deemed necessary to meet the participants’ needs at the time the survey was given, and whether or not those accommodations were requested and/or received). Data missingness is generally low in our study, likely in part because our survey was administered in a clinical setting (the waiting area of an OB/GYN clinic). Still, some models in our analyses had slight missingness, and like most epidemiological studies, we cannot rule out the possibility that missing data might introduce additional bias into the observed results. The cross-sectional nature of the survey makes it difficult to establish inferences about causality for some of the significant predictors identified, such as job satisfaction and satisfaction with supervisor/manager. Pregnant employees who were dissatisfied with their job or supervisor/manager had lower odds of receiving certain accommodations, which may indicate that the climate at work or working relationship with their supervisor/manager discourages negotiation about accommodations. Conversely, not receiving accommodations conducive to better work performance may decrease satisfaction with a job or supervisor/manager. There is also potential for unmeasured confounding. We also recognize that workplace exposures do not occur in isolation of each other; perhaps a future study can account for exposure mixtures in the analyses, determine a composite measure of occupational risk, and examine how it affects PWFA awareness and receipt of reasonable accommodations. Finally, the low prevalence of PWFA awareness may place limits on statistical power.

Future studies with larger sample sizes may consider examining the multiple-way interactions among different demographic and occupational factors. Our study was conducted in a hospital that serves a lower socioeconomic status community in NYC, which may not fully represent the pregnant working population covered by the NYC PWFA. We recommend exercising caution when generalizing these results beyond New York City due to its ethnically diverse population and the unique legal landscape of local laws and policies protecting pregnant workers and their families. In light of the national PWFA legislation that went into effect in June 2023, our study provides timely and valuable information on the factors associated with PWFA awareness and utilization in New York City before the national legislation. It would be insightful to replicate this study’s framework and investigate how much has changed for pregnant workers in New York City after the federal law was passed.

In summary, our study reflects that the PWFA law was virtually unknown and thus critically underutilized within the first five years of its enactment. Women born outside of the U.S., with fewer years of education, shorter job tenure, lack of a paid workplace maternity leave policy, and lack of private health insurance had lower odds of receiving PWFA-eligible accommodations relative to their counterparts. As more women continue to work during pregnancy, interventions promoting PWFA awareness and utilization are particularly important for protecting maternal & child health. The conversation surrounding pregnant workers’ rights is crucial, especially after the national PWFA went into effect across the U.S. in June 2023, and the Equal Employment Opportunity Commission issued final regulations that became effective in June 2024. The implementation of a federal PWFA also warrants follow-up studies, as legislation on a national platform may draw greater attention to this topic and drive more pregnant workers to initiate negotiations about reasonable accommodations in the workplace. We recommend future studies investigate how knowledge about PWFA legislation can be distributed more effectively in the workplace, so that all pregnant workers and their families can benefit from it.

## Electronic supplementary material

Below is the link to the electronic supplementary material.

Supplementary Material 1: **Figure S1**. Flowchart illustrating the structure of the questionnaire instrument used to collect data on participants’ awareness and understanding of the PWFA law, as well as accommodations received regardless of PWFA awareness. The questionnaire was set up on REDCap/iPad with branching logics and cannot backtrack after answering a question. **Table S1**. Results from the count and logit components of the multivariable-adjusted zero-inflated Poisson regression model. The model examines the sociodemographic and occupational factors associated with lacking knowledge of PWFA-eligible accommodations. The dependent variable (outcome) is the number of PWFA-eligible accommodations recognized by a participant (discrete count data, from 0 to 6). The adjusted odds ratio (aOR) from the logit component of the zero-inflated Poisson regression model represents the odds of recognizing zero (none) of the eligible accommodations (i.e., did not recognize any of the six examples of accommodations as PWFA-eligible), compared to the reference group (for categorical predictors) or corresponding to per 1-unit increase in the predictor (for continuous variables such as age). The count component of the model (i.e., Poisson process) derives the effect estimates (β) of the Poisson regression given the outcome is not an excess zero (i.e., recognizing ≥1 PWFA-eligible accommodations); the β estimate represents the change in the expected count of the outcome, compared to the reference group (for categorical predictors) or corresponding to per 1-unit increase in the predictor (for continuous variables). **Appendix A** - ESPWFA Study Survey (English ver.). English language version of the ESPWFA pilot project pregnancy & work survey.

## Data Availability

The datasets used and analyzed during the current study can be made available from the corresponding author upon reasonable request.

## Abbreviations

NYC: New York City
PWFA: Pregnant Workers Fairness Act
OR: Odds Ratio
aOR: Adjusted Odds Ratio
CI: Confidence Interval
U.S.: United States
PDA: Pregnancy Discrimination Act of 1978
ADAAA: Americans with Disabilities Act Amendments Act of 2008
ESPWFA: Effectiveness Study of Pregnant Workers Fairness Act
EEOC: Equal Employment Opportunity Commission
