# Supplementary material for: Sociodemographic and occupational factors influencing pregnant workers’ awareness and utilization of the New York City Pregnant Workers Fairness Act
Source: BMC Public Health. 2025 Aug 1;25:2606. doi: 10.1186/s12889-025-23404-w (PMC12315298; doi:10.1186/s12889-025-23404-w)
Supplement: Supplementary file 1 — Supplementary Material 1: Figure S1. Flowchart illustrating the structure of the questionnaire instrument used to collect data on participants’ awareness and understanding of the PWFA law, as well as accommodations received regardless of PWFA awareness. The questionnaire was set up on REDCap/iPad with branching logics and cannot backtrack after answering a question. Table S1. Results from the count and logit components of the multivariable-adjusted zero-inflated Poisson regression model. The model examines the sociodemographic and occupational factors associated with lacking knowledge of PWFA-eligible accommodations. The dependent variable (outcome) is the number of PWFA-eligible accommodations recognized by a participant (discrete count data, from 0 to 6). The adjusted odds ratio (aOR) from the logit component of the zero-inflated Poisson regression model represents the odds of recognizing zero (none) of the eligible accommodations (i.e., did not recognize any of the six examples of accommodations as PWFA-eligible), compared to the reference group (for categorical predictors) or corresponding to per 1-unit increase in the predictor (for continuous variables such as age). The count component of the model (i.e., Poisson process) derives the effect estimates (β) of the Poisson regression given the outcome is not an excess zero (i.e., recognizing ≥1 PWFA-eligible accommodations); the β estimate represents the change in the expected count of the outcome, compared to the reference group (for categorical predictors) or corresponding to per 1-unit increase in the predictor (for continuous variables). Appendix A - ESPWFA Study Survey (English ver.). English language version of the ESPWFA pilot project pregnancy & work survey. [file 12889_2025_23404_MOESM1_ESM.pdf]

Supplemental Materials

Figure S1. Flowchart illustrating the structure of the questionnaire instrument used to collect data on participants' awareness and understanding of the PWFA law, as well as accommodations received regardless of PWFA awareness. The questionnaire was set up on REDCap/iPad with branching logics and cannot backtrack after answering a question.

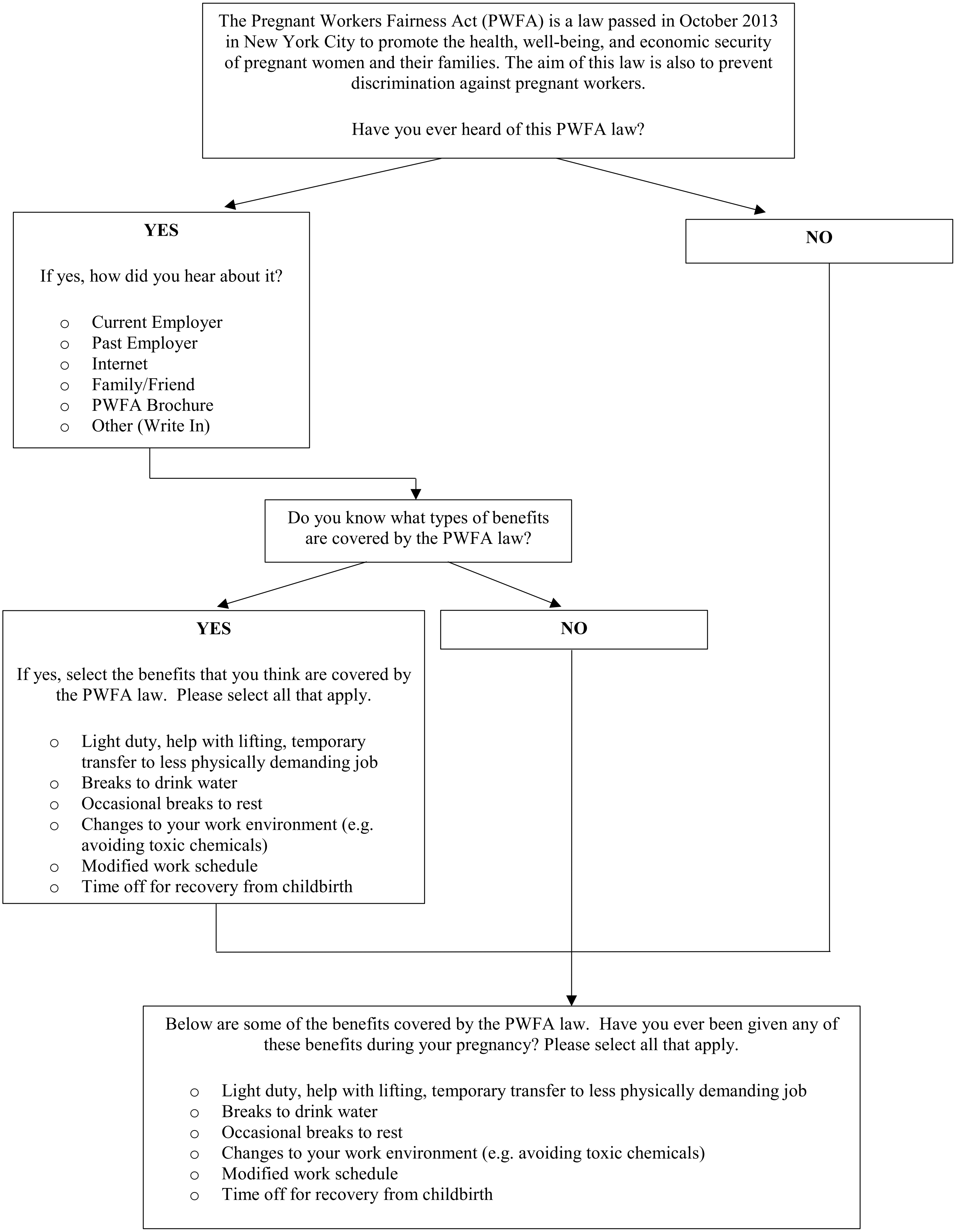

Table S1. Results from the count and logit components of the multivariable-adjusted zero-inflated Poisson regression model. The model examines the sociodemographic and occupational factors associated with lacking knowledge of PWFA-eligible accommodations. The dependent variable (outcome) is the number of PWFA-eligible accommodations recognized by a participant (discrete count data, from 0 to 6). The adjusted odds ratio (aOR) from the logit component of the zero-inflated Poisson regression model represents the odds of recognizing zero (none) of the eligible accommodations (i.e., did not recognize any of the six examples of accommodations as PWFA-eligible), compared to the reference group (for categorical predictors) or corresponding to per 1-unit increase in the predictor (for continuous variables such as age). The count component of the model (i.e., Poisson process) derives the effect estimates (β) of the Poisson regression given the outcome is not an excess zero (i.e., recognizing ≥1 PWFA-eligible accommodations); the β estimate represents the change in the expected count of the outcome, compared to the reference group (for categorical predictors) or corresponding to per 1-unit increase in the predictor (for continuous variables).

**Characteristics**	**Count component**		**Logit component**	
	**β (95% CI)**	***p*-value**	**aOR (95% CI)**	***p*-value**
**Age (in years)**	-0.01 (-0.04, 0.02)	0.63	0.97 (0.90, 1.03)	0.32
**Race/Ethnicity**				
White	Ref.	--	Ref.	--
Black & Hispanic	-0.18 (-0.73, 0.37)	0.52	1.1 (0.42, 2.91)	0.85
**Education**				
>12th grade	Ref.	--	Ref.	--
≤12th grade	-0.27 (-0.75, 0.22)	0.28	1.56 (0.72, 3.39)	0.26
**Maternity leave policy**				
Paid	Ref.	--	Ref.	--
Unpaid	0.26 (-0.42, 0.93)	0.46	1.91 (0.76, 4.76)	0.17
No policy	-0.12 (-0.90, 0.67)	0.77	6.83 (2.07, 22.58)	0.002
Unsure	0.24 (-0.45, 0.93)	0.49	5.47 (1.74, 17.22)	0.004
**High-risk pregnancy clinic**				
No	Ref.	--	Ref.	--
Yes	-0.47 (-1.06, 0.13)	0.13	1.92 (0.71, 5.14)	0.20
**Supervisor/manager status**				
Yes	Ref.	--	Ref.	--
No	-0.15 (-0.62, 0.32)	0.53	2.92 (1.29, 6.60)	0.01

Multivariable model adjusted for all characteristics shown in table, as well as marital status, job satisfaction, satisfaction with supervisor/manager, country born, and job tenure; *n*=475.

# - Appendix A -
ESPWFA Study Survey (English ver.)

Part A: Basic Information

Please answer the following questions to the best of your knowledge.

****

A1. What is your current age (in years)?

______________________ (years)

A2. How would you best describe your race or ethnicity? (Please select ALL that apply)

☐ White ☐ Black ☐ Hispanic or Latino ☐ Native American ☐ Asian / Pacific Islander
☐ Other

______________________ (If "Other", please specify.)

A3. What is your marital status?

◯ Married
◯ Not married, but living with a partner
◯ Single, never married
◯ Divorced, separated, or widowed
◯ Other

______________________ (If "Other", please specify.)

A4. Until now, how many times did you give birth?

◯ 0 ◯ 1 ◯ 2 ◯ 3
◯ 4 ◯ 5 or more
(time(s))

A5. How many children (including stepchildren) do you have?

◯ 0 ◯ 1 ◯ 2 ◯ 3
◯ 4 ◯ 5 or more
(children)

--A5a1. How many of your children are ages 0-11 years old?

◯ 0 ◯ 1 ◯ 2 ◯ 3 or more
(children)

--A5a2. How many of your children are ages 12-18 years old?

◯ 0 ◯ 1 ◯ 2 ◯ 3 or more
(children)

--A5a3. How many of your children are age 19 years old or older?

◯ 0 ◯ 1 ◯ 2 ◯ 3 or more
(children)

A6. How many people (NOT including yourself) do you financially support now?

◯ 0 ◯ 1 ◯ 2 ◯ 3
◯ 4 ◯ 5 or more
(person(s))

A7. Are you the only person in your home financially supporting your family?

◯ Yes ◯ No

A8. What is your total household yearly income during the past year (from ALL people who contribute to it)?

◯ Less than $20,000 (less than $1,667 monthly)
◯ $20,000 to $29,999 ($1,667 to $2,499 monthly)
◯ $30,000 to $49,999 ($2,450 to $4,166 monthly)
◯ $50,000 to $99,999 ($4,167 to $8,333 monthly)
◯ $100,000 or more ($8,334 monthly or more)

A9. What is the highest degree or level of school you have completed?

- ◯ Did not finish high school (less than 12th grade)
- ◯ High school diploma or GED
- ◯ Trade/technical/vocational training, some college (less than 4 years), or Associate's degree
- ◯ Bachelor's degree (4 years of college)
- ◯ Graduate degree (Master's or doctoral/professional degree such as MD, PhD, JD, etc.)

A10. What is the main language you speak at home?

◯ English ◯ Spanish
◯ Mandarin ◯ Other

____
(If "Other", please specify.)

A11. In what country were you born?

- ◯ United States (U.S.)
- ◯ Other country

____
(If "Other country", please specify.)

A12. Where do you currently live?

- ◯ New York City (Brooklyn, Bronx, Manhattan, Queens, Staten Island)
- ◯ Long Island (Nassau or Suffolk counties)
- ◯ Other New York State (NOT including New York City or Long Island)
- ◯ New Jersey
- ◯ Other

____
(If "Other", please specify.)

A13. Have you ever smoked regularly (1 or more cigarettes/cigars/pipes, etc. per day)?

◯ Yes ◯ No

--A13a. Do you consider yourself a regular smoker now?

◯ Yes ◯ No

--A13b. If yes, how many cigarettes do you smoke a day?

____
(cigarette(s))

A14. Do you consume alcohol on a regular basis?

◯ Yes ◯ No

--A14a. If yes, how many drink(s) a day? (1 drink is a 12-oz beer, or 6-oz wine, or 1-oz liquor)

- ◯ 1
- ◯ 2
- ◯ 3
- ◯ 4
- ◯ 5 or more

(drink(s))

A15. What is your height?

- ◯ 4 ft
- ◯ 5 ft
- ◯ 6 ft
- ◯ 7 ft

(feet; ft)

◯ 0 in
◯ 1 in
◯ 2 in
◯ 3 in
◯ 4 in
◯ 5 in
◯ 6 in
◯ 7 in
◯ 8 in
◯ 9 in
◯ 10 in
◯ 11 in
(inches; in)

A16. What was your weight before pregnancy (in pounds)?

____
(pounds; lb)

A17. How would you describe your overall health?

◯ Excellent ◯ Very good ◯ Good ◯ Fair ◯ Poor

A18. What type of health insurance do you currently have?

◯ I do not have health insurance
◯ Medicaid
◯ Medicare
◯ Private insurance
◯ Medicare and private insurance
◯ Other

A19. Are you a patient in our high-risk pregnancy clinic?

◯ Yes ◯ No

--A19a. If yes, why are you being followed in the high-risk clinic? (Please select ALL that apply.)

☐ Asthma
☐ High blood pressure, diagnosed DURING pregnancy
☐ High blood pressure, diagnosed BEFORE pregnancy
☐ Diabetes, diagnosed DURING pregnancy
☐ Diabetes, diagnosed BEFORE pregnancy
☐ Other health condition affecting me
☐ Other health condition affecting my baby

A20. As you are having a baby, we would like to know how you are feeling. Please mark the answer which comes closest to how you have felt IN THE PAST 7 DAYS - not just how you feel today.

In the past 7 days...

	Most of the Time	Some of the Time	Not Very Often	Not at All
I have been able to laugh and see the funny side of things	○	○	○	○
I have looked forward with enjoyment to things	○	○	○	○
I have blamed myself unnecessarily when things went wrong	○	○	○	○
I have been anxious or worried for no good reason	○	○	○	○
I have felt scared or panicky for no good reason	○	○	○	○
Things have been overwhelming me (getting on top of me)	○	○	○	○
I have been so unhappy that I have had difficulty sleeping	○	○	○	○
I have felt sad or miserable	○	○	○	○
I have been so unhappy that I have been crying	○	○	○	○
The thought of harming myself has occurred to me	○	○	○	○

Part B: Job Information

Please answer the following questions to the best of your knowledge.

****

B1. How many jobs have you held since January 2014?

◯ 0 ◯ 1 ◯ 2 ◯ 3 ◯ 4 ◯ 5 or more

B2. How many jobs do you currently have?

◯ 0 ◯ 1 ◯ 2 ◯ 3 or more

The following questions will be based on your MAIN job in New York City.

B3. Where do you work?

◯ Brooklyn ◯ Bronx ◯ Manhattan ◯ Queens ◯ Staten Island ◯ Other

____
(If "Other", please specify.)

B4. Are there four or more people working for your employer?

◯ Yes ◯ No

B5. What is your main job title?

____

B6. What industry/field is your job in? (Pick one that describes it the best)

◯ Agriculture, Food and Natural Resources
◯ Architecture and Construction
◯ Arts, Audio/Video Technology & Communications
◯ Business, Finance & Marketing
◯ Education & Training
◯ Government & Public Administration
◯ Health Care (Medicine, Nursing, etc.)
◯ Hospitality & Tourism
◯ Human Services
◯ Law, Public Safety, Corrections, & Security
◯ Manufacturing
◯ Science, Technology, Engineering, & Math
◯ Transportation, Distribution, & Logistics
◯ Other

____
(If "Other", please specify.)

B7. Are you a supervisor/manager at your worksite?

◯ Yes ◯ No

If you are currently pregnant, please answer the following questions based on THIS pregnancy. If you recently gave birth, please answer the following questions based on your MOST RECENT pregnancy.

B8. BEFORE this pregnancy or your most recent pregnancy, which of the following activities did you do at work? Check all that apply.

☐ Standing
☐ Sitting
☐ Walking
☐ Lifting
☐ Pushing or pulling heavy objects
☐ Other

____
(If "Other", please specify.)

--B8a. BEFORE this pregnancy or your most recent pregnancy, how many hours a day did you stand at work?

____
(hour(s))

--B8b. BEFORE this pregnancy or your most recent pregnancy, how many hours a day did you sit at work?

____
(hour(s))

--B8c. BEFORE this pregnancy or your most recent pregnancy, how many hours a day did you walk at work?

____
(hour(s))

--B8d. BEFORE this pregnancy or your most recent pregnancy, how many hours a day did you lift objects at work?

____
(hour(s))

--B8e. BEFORE this pregnancy or your most recent pregnancy, how many hours a day did you push or pull heavy objects at work?

____
(hour(s))

--B8f. BEFORE this pregnancy or your most recent pregnancy, how many hours a day did you perform the activity listed as "Other" in question B8?

____
(hour(s))

B9. Please rate your overall physical effort at the job BEFORE this pregnancy or your most recent pregnancy.

○ Very hard ○ Hard ○ Somewhat hard ○ Fairly light ○ Very light

B10. DURING this pregnancy or your most recent pregnancy, which of the following activities do you do at work? Check all that apply.

☐ Standing
☐ Sitting
☐ Walking
☐ Lifting
☐ Pushing or pulling heavy objects
☐ Other
☐ Did not work during pregnancy

____
(If "Other", please specify.)

--B10a. DURING this pregnancy or your most recent pregnancy, how many hours a day do you stand at work?

____
(hours)

--B10b. DURING this pregnancy or your most recent pregnancy, how many hours a day do you sit at work?

____
(hour(s))

--B10c. DURING this pregnancy or your most recent pregnancy, how many hours a day do you walk at work?

____
(hour(s))

--B10d. DURING this pregnancy or your most recent pregnancy, how many hours a day do you lift objects at work?

____
(hour(s))

--B10e. DURING this pregnancy or your most recent pregnancy, how many hours a day do you push or pull heavy objects at work?

__________________ (hour(s))

--B10f. DURING this pregnancy or your most recent pregnancy, how many hours a day do you perform the activity listed as "Other" in question B10?

__________________ (hour(s))

B11. Please rate your overall physical effort at the job DURING this pregnancy or your most recent pregnancy.

○ Very hard ○ Hard ○ Somewhat hard ○ Fairly light ○ Very light ○ Did not work during pregnancy

B12. BEFORE this pregnancy or your most recent pregnancy, were you exposed to any chemicals in your workplace?

○ Yes ○ No

--B12a. If yes, which of the following chemicals were you exposed to in the workplace? (Please check ALL that apply.)

☐ Cleaning chemicals
☐ Pesticides
☐ Paints/Sprays
☐ Metals
☐ Other chemical(s)

__________________ (If "Other", please specify.)

--B12b. How many hours per day were you exposed to these chemicals?

__________________ (hour(s) per day)

B13. DURING this pregnancy or your most recent pregnancy, are you exposed to any chemicals in your workplace?

○ Yes ○ No ○ Did not work during pregnancy

--B13a. If yes, which of the following chemicals are you exposed to in the workplace? (Please check ALL that apply.)

☐ Cleaning chemicals
☐ Pesticides
☐ Paints/Sprays
☐ Metals
☐ Other chemical(s)

__________________ (If "Other", please specify.)

--B13b. How many hours per day are you exposed to these chemicals?

__________________ (hour(s) per day)

B14. What is your USUAL shift for this job?

○ Day shift
○ Evening shift (beginning at/or after 5pm)
○ Late night shift (beginning at/or after 10pm)
○ My work shifts often change

B15. How many hours a week do you normally work at this job?

__________________ (hour(s) per week)

--B15a. Is this a full-time job or part-time job?

○ full-time
○ part-time

B16. How long have you been working at this job?

○ less than 6 months
○ 6 months-1 year
○ 1-2 yr
○ 2-5 yr
○ more than 5 years

B17. Does your job have a maternity leave policy?

◯ Yes, paid maternity leave ◯ Yes, but not paid ◯ No ◯ Not sure

B18. How satisfied are you with your job?

◯ Very satisfied ◯ Somewhat satisfied ◯ Not too satisifed ◯ Not satisfied at all

B19. How satisfied are you regarding your work relationship with your supervisor/boss?

◯ Very Satisfied ◯ Somewhat Satisfied ◯ Not too Satisfied ◯ Not satisfied at all

B20. Do you feel stressed at this job?

◯ Very stressed ◯ Somewhat stressed ◯ Not too stressed ◯ Not stressed at all

B21. Have you ever left your job because of this pregnancy or previous pregnancies?

◯ Yes ◯ No

--B21a. If yes, what was the main reason you left your job?

◯ My employer asked me to leave the job after I got pregnant.
◯ I decided to leave the job because I did not want to work when I was pregnant.
◯ I decided to leave the job because it was difficult for me to do my job while pregnant.

B22. What is the main language spoken in your workplace?

◯ English ◯ Spanish ◯ Mandarin ◯ Other

____
(If "Other", please specify.)

Part C: Pregnant Workers Fairness Act

Please answer the following questions to the best of your knowledge.

****

This section includes questions on your familiarity with the Pregnant Workers Fairness Act (PWFA).

The Pregnant Workers Fairness Act (PWFA) is a law passed in 2013 to promote the health, well-being, and economic security for pregnant women and their families. The aim of this law is also to prevent discrimination against pregnant workers.

Next, we will ask you a few questions about this law.

C1. Have you ever heard of this PWFA law?

○ Yes ○ No

--C1a. If yes, where did you hear about it? Please select ALL that apply.

☐ Current Employer ☐ Past Employer ☐ Internet ☐ Family/Friend ☐ PWFA Brochure
☐ Other

____
(If "Other", please specify.)

----C1a1. How were you informed by your employer about the PWFA law? Please select ALL that apply.

☐ Email ☐ Poster/Brochure ☐ Word of Mouth ☐ Other

____
(If "Other", please specify)

----C1a2. When were you informed by your employer about the PWFA law?

○ Before 2014 ○ 2014 ○ 2015 ○ 2016-2017 ○ Do not remember

The Pregnant Workers Fairness Act (PWFA) is a law passed in 2013 to promote the health, well-being, and economic security for pregnant women and their families. The aim of this law is also to prevent discrimination against pregnant workers.

C2. Do you know that your employer is required to inform you about the PWFA law?

◯ Yes ◯ No

C3. Do you know what benefits are covered by the PWFA law?

◯ Yes ◯ No

--C3a. If yes, select the benefits below that you are aware of: Please select ALL that apply.

- ☐ Light duty, help with lifting, temporary transfer to less physically demanding job
- ☐ Breaks to drink water
- ☐ Occasional breaks to rest
- ☐ Changes to your work environment (e.g. avoiding toxic chemicals)
- ☐ Modified work schedule
- ☐ Time off for recovery from childbirth

--C3b. If yes (to C3), which group of people is covered by the PWFA law? Please select ALL that apply.

- ☐ Employees who are pregnant
- ☐ Employees who just gave birth
- ☐ Employees who have pregnancy or childbirth related conditions

The Pregnant Workers Fairness Act (PWFA) is a law passed in 2013 to promote the health, well-being, and economic security for pregnant women and their families. The aim of this law is also to prevent discrimination against pregnant workers.

C4. Below are some of the benefits covered by the PWFA law. Have you been given any of these benefits during your pregnancy? Please select ALL that apply.

- ☐ Light duty, help with lifting, temporary transfer to less physically demanding job
- ☐ Breaks to drink water
- ☐ Occasional breaks to rest
- ☐ Time off for recovery from childbirth
- ☐ Changes to your work environment (e.g. avoiding toxic chemicals)
- ☐ Modified work schedule
- ☐ I have not been given any of these benefits listed above

C5. Do you know that your employer is required by law to provide these work benefits during and/or after your pregnancy?

◯ Yes ◯ No

C6. Are you comfortable asking your employer to give you these work benefits during pregnancy?

◯ Yes ◯ No

C7. Do you think that the Pregnant Workers Fairness Act (PWFA) law will help to keep you healthy at work during your pregnancy?

◯ Yes, Strongly Agree ◯ Yes, Agree ◯ Neutral ◯ No, Disagree ◯ No, Strongly Disagree
